# Bridging the digital divide: the association between smart health devices and disability risk among digitally marginalized older adults in China

**DOI:** 10.1186/s12939-026-02766-6

**Published:** 2026-01-20

**Authors:** Zhang Chi, LongXuan Lin, Wang Li, Han Hu

**Affiliations:** 1 School of Philosophy and Sociology, Lanzhou University, LanZhou, China; 2 School of Philosophy and Sociology, Northwest Normal University, LanZhou, China; 3 School of Public Policy and Administration, Xi’an Jiaotong University, Xi’an, China

**Keywords:** Smart health devices, Risk of incapacitation, Older adults with incapacitation, Impact mechanisms

## Abstract

**Background:**

This study explores the association of smart health devices on the risk of disability among older adults, and the underlying pathways. The study is grounded in three key factors: the reality of China’s rapidly developing ageing process; the popularisation of smart health devices; and the rapid development of the digital economy.

**Methods:**

This study utilised the CLASS2020 dataset to investigate the association of smart health devices on the risk of disability among older adults. Multiple linear regression was employed to analyse the data, and a robustness test was conducted by shrinking the sample using propensity score matching. This study utilised the Sobel and bootstrap methods to analyse the mediating effect of smart health devices on the risk of incapacitation among older adults. Additionally, it examined the heterogeneity of digital access and literacy in relation to the association of smart health devices on the risk of incapacitation among older adults.

**Results:**

The findings of this study indicated that the implementation of smart health devices, as well as the quantity of these devices utilized by older adults, exhibited a negative association on the risk of disability in this demographic. The analysis of mediating pathways suggested that the association between smart health device use and lower disability risk was partially explained by its correlation with higher levels of intergenerational financial and household support, greater exercise frequency, and increased use of rehabilitation therapy. The analysis of moderating effects demonstrated that older adults' comparative health level exerted a negative moderating effect, while recent hospitalization experience exerted a positive moderating effect.

**Conclusion:**

The negative association of smart health devices on the risk of disability in older individuals is more pronounced among those without digital access and with limited digital literacy, while its efficacy is diminished among those with digital access and proficient digital literacy. If the observed associations are causal, our findings suggest that the government promote the universalization of smart health devices, further bridge the digital divide among the older adults, and enhance the digital literacy of the older adults through digital education.

## Introduction

The progression of the global ageing trend has given rise to a significant issue in the process of ageing across all countries: disability in older persons. The phenomenon of global population ageing has given rise to a considerable increase in health problems, including chronic diseases, motor system deterioration and cognitive impairment [1]. As indicated by data provided by the Ministry of Civil Affairs, the number of disabled older adults in China was approximately 35 million in September 2024, constituting 11.6% of the total population of older adults in the country [2]. It is anticipated that the population will increase to 91.4 million by the year 2050 [3]. A shift in the disease spectrum of older adults in China has been observed, with chronic diseases such as hypertension, diabetes and heart disease becoming the leading cause of health problems among this demographic. This increase in chronic diseases is likely to result in an elevated risk of disability among older adults [4].

In order to address the issue of disability among the older adults and enhance their quality of life and health, the Chinese government has implemented a series of pertinent policies. The Chinese government has proposed a strategy for the provision of senior care services that is both precise and personalised, with the 14th Five-Year Plan explicitly proposing the establishment of a senior care service network that is “accurate, personalised, and specialized”. The strategy is intended to optimise the allocation of resources by means of a dynamic evaluation of the level of disability, income level, and family structure of older adults [5]. The State Council has established a target of ensuring comprehensive coverage of geriatric departments in general hospitals at the second level and above by the year 2025. Furthermore, the Council is promoting the signing of contracts and cooperation between medical institutions and institutions specializing in the care of older adults. The “medical and nursing consortium” model in Zhejiang and Shandong has embedded chronic disease management and rehabilitation training within the community. This facilitates the provision of professional care to older adults and individuals with disabilities in close proximity to their place of residence [6, 7].

The advent of the digital economy, coupled with the gradual proliferation of smart health devices [8, 9], has engendered a paradigm shift in the manner in which the Chinese government has approached the provision of care services for older adults. This shift has been precipitated by the government’s ability to leverage technological advancements to enhance the well-being of this demographic. In an effort to mitigate the risk of incapacitation, Chongqing, Sichuan, and other regions have initiated a pilot program that aims to digitally empower older adults by employing advanced technologies such as smart aging and 5G-based remote monitoring systems [10, 11]. While the extant literature provides valuable insights, it exhibits three primary limitations that this study seeks to address. First, conceptually, prior studies have often focused on the impact of a single type of smart health device in isolation, failing to capture the aggregate effect of multiple devices that an older adult might use. Second, methodologically, there is a scarcity of research that utilizes large-scale, nationally representative data to empirically test the direct association between smart health device adoption and disability risk. Third, and most critically, the underlying mechanisms—the how and for whom—remain underexplored. A comprehensive investigation that simultaneously examines mediating pathways and moderating effects is lacking. This study fills these conceptual and methodological gaps by utilizing the national CLASS2020 dataset to investigate the holistic association of smart health device use on disability risk and to empirically test an integrated theoretical framework encompassing behavioral change, social support, and health capital theories. The study, which was based on the CLASS2020 dataset, employed multiple linear regression modeling to investigate the association of smart health devices on the risk of disability experienced by older adults and the potential pathways explaining this association. Guided by the aforementioned gaps in the literature, this study is driven by the following overarching research question: What is the association between smart health device adoption and the risk of disability among digitally marginalized older adults in China, and what are the key behavioral, social, and health-related mechanisms that explain and moderate this relationship?

## Literature and theoretical framework

### Smart health devices and older adults’ health: a fragmented landscape

Existing research suggests that smart health devices can positively influence health management and disease prevention among older adults. Devices such as smart bracelets and voice assistants facilitate proactive health management through real-time monitoring of physiological indicators (e.g., heart rate, steps) and health reminders (e.g., for medication) [12, 13]. For instance, the feedback from smart bracelets can enhance health awareness, motivating preventive behaviors that may mitigate disability risk from chronic diseases [14]. Furthermore, wearable activity trackers have been shown to promote physical activity, improve ambulatory function, and reduce sedentary behavior, directly contributing to maintained physical capability and reduced fall risk [12, 13]. Beyond physical health, these devices may also improve mental well-being and social connectedness by reducing loneliness through access to social activities and health information, which is critically linked to disability risk [15].

However, the current body of evidence remains fragmented. The majority of studies focus on the impact of a single type of device (e.g., only smart bracelets) or are conducted within specific, limited regional contexts. This singular focus fails to capture the real-world scenario where older adults may own or use multiple devices simultaneously, creating a conceptual gap in understanding the aggregate effect of smart health device adoption. Moreover, there is a methodological scarcity of large-scale, nationally representative studies that empirically test the direct link between device usage and disability risk. Most critically, the underlying mechanisms—the precise pathways through which these devices exert their influence—remain a black box. While potential mediators like exercise or social support are often suggested, they are seldom tested within an integrated model that also accounts for key moderating factors.

### Theoretical foundations and hypothesis development

To address the complex interplay between technology, behavior, and health outcomes, this study is grounded in an integrated multi-theoretical framework. We synthesize four theoretical perspectives to provide a comprehensive explanation of how smart health devices might influence disability risk (mediating mechanisms), for whom this influence is most potent (moderating effects), and the critical precondition of technology adoption itself.

First, the Health Belief Model (HBM) [16] posits that health behaviors are influenced by perceived susceptibility, benefits, and cues to action. We conceptualize smart health devices as persistent, personalized cues that enhance awareness of health status, thereby motivating engagement in protective behaviors such as physical activity and rehabilitation; Second, Social Support Theory [17], particularly its “buffering” model, suggests that resources from social networks can mitigate the adverse effects of stressors like health decline. For older adults in China, intergenerational support from children is a paramount resource. Smart health devices, through features like remote monitoring and communication, can facilitate the flow of instrumental and financial support, potentially buffering against disability risk; Third, Health Capital Theory (HCT) [18] frames health as a stock that depreciates with age but can be augmented through investments. The use of a smart health device is conceptualized as a form of health investment. This theory predicts that the return on this investment—the reduction in disability risk—may be contingent on an individual’s pre-existing health capital; Finally, and crucially, the Technology Acceptance Model (TAM) [19] provides the foundational logic for the adoption and use of the technology itself. It asserts that actual usage is determined by perceived usefulness and perceived ease of use. For digitally marginalized older adults, barriers to either perception can prevent engagement with the very devices that the other theories hypothesize will confer health benefits. Thus, TAM is not an ancillary add-on but a core component of our framework, explaining the initial hurdle of technology engagement upon which the behavioral, social, and investment mechanisms depend.

Collectively, these theories suggest a sequential yet interconnected process: the adoption and use of devices (explained by TAM) enable them to function as cues to action (HBM) and facilitators of social support (Social Support Theory), which collectively represent a health investment (HCT) whose efficacy is moderated by baseline health capital. This integrated logic leads to our foundational hypothesis:

#### H1

Smart health device use is associated with a lower risk of disability in older adults.

To unpack the mechanisms underlying H1, we draw on HBM and Social Support Theory to specify mediating pathways:

#### H2

The negative association between smart health devices and disability risk is partially mediated by (a) increased exercise frequency and (b) increased use of rehabilitation therapy.

#### H3

The negative association between smart health devices and disability risk is partially mediated by (a) increased intergenerational financial support and (b) increased intergenerational household support.

To understand for whom this association is strongest, HCT guides our examination of health-related moderators:

#### H4

The negative association between smart health devices and disability risk is moderated by health capital, such that it is stronger among those with (a) poorer comparative health, (b) recent hospitalization experience, and (c) a greater number of chronic diseases.

Furthermore, based on TAM, we formally hypothesize that the core relationships in our model are not uniform but are contingent on the digital context of the user:

**H5**: (TAM-based heterogeneity): The negative association between smart health devices and disability risk is stronger for older adults facing a digital divide and with lower digital literacy, as these groups stand to gain a greater marginal benefit from successful technology adoption.

### Synthesizing the gaps and this study’s contribution

**Fig. 1:**
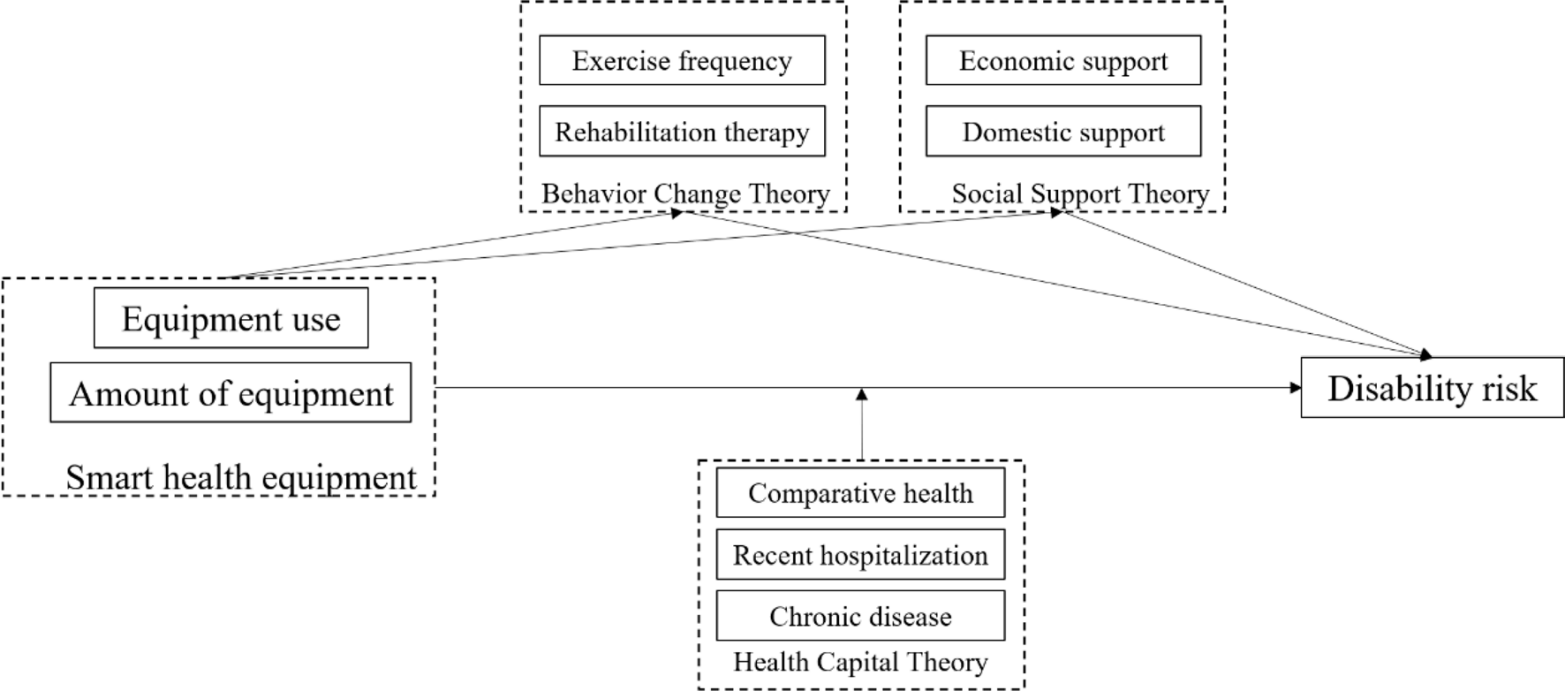
Research framework for smart health devices on risk of disability in older adults

In synthesis, the literature reveals a clear need for a unified analysis that moves beyond examining devices in isolation to model the complex interplay between device usage, its behavioral and social pathways, and individual health contingencies. To address the conceptual gap of a fragmented device focus, our study investigates both the use and the number of smart health devices, first testing the overall association (H1). To tackle the methodological gap, we employ a large-scale, national dataset (CLASS2020). Most importantly, to illuminate the mechanistic black box, we propose and test a comprehensive model (Fig. 1) that integrates mediators from the HBM and Social Support Theory with moderators from Health Capital Theory.

The research framework is visually summarized in Fig. 1. It posits that smart health device usage (the independent variable, measured both in terms of adoption and quantity) is associated with a lower risk of disability (the dependent variable). This main relationship (H1) is hypothesized to operate through four mediating pathways (H2a, H2b, H3a, H3b): increased exercise frequency, greater use of rehabilitation therapy, and enhanced intergenerational financial and household support. Furthermore, the model specifies that this relationship is moderated by three factors related to an individual’s health capital (H4a, H4b, H4c): comparative health level, recent hospitalization experience, and the number of chronic diseases. By simultaneously testing the foundational association (H1) and its underlying mediating and moderating mechanisms (H2-H4), our research provides a more nuanced understanding. It allows us to not only confirm whether an association exists but also to explain how it works and for whom it is most effective, thereby offering deeper insights for both theory and policy development in smart aging.

## Data, variables and models

### Data

The China Longitudinal Aging Social Survey (CLASS) is a nationally representative longitudinal survey designed to systematically investigate the social, economic, and health conditions of the elderly population in China. Conducted by the National Survey Research Center at Renmin University of China, the survey employs a sophisticated sampling strategy to ensure the collection of high-quality, scientifically valid data that accurately reflects the circumstances of older adults across the country. The sampling scheme for CLASS is a stratified, multi-stage probability proportional to size (PPS) design, which effectively captures the socioeconomic and geographical diversity of China’s aging population. The process begins with the stratification of all county-level units (including both urban districts and rural counties) across 28 provinces, autonomous regions, and municipalities. These strata are constructed based on regional socioeconomic development indicators and urbanization levels to ensure proportional representation of different regional characteristics. The first sampling stage involves the random selection of primary sampling units (PSUs) from these stratified county-level units, with probability proportional to their population size of elderly residents. In the second stage, villages and urban neighborhoods are selected as secondary sampling units within each chosen PSU, again using PPS sampling based on the concentration of elderly residents. The final sampling stage employs a meticulous mapping and listing procedure to identify all eligible households within these selected communities. This approach is particularly crucial in the Chinese context, where traditional sampling frames based on household registration records often fail to account for substantial internal migration and complex living arrangements. Trained enumerators conduct comprehensive community mappings, creating detailed residential inventories that serve as the sampling frame for household selection. The target population consists of Chinese citizens aged 60 years or older who reside in private households within the sampled communities. The baseline survey, conducted in 2014, successfully interviewed 11,511 eligible respondents from 462 villages/neighborhoods across 134 county-level units, achieving a well-balanced representation of both urban and rural elderly populations. The longitudinal nature of CLASS enables researchers to track individual-level changes over time, with follow-up surveys conducted approximately every two years to monitor dynamic processes in aging, including health transitions, economic status fluctuations, and evolving social support networks.

The initial CLASS2020 dataset contained 11,511 observations. It is important to note that the CLASS survey employs a long-form and short-form questionnaire design. Our study’s analytical focus on disability risk required variables (e.g., detailed ADL, IADL, and cognitive assessments) that were primarily contained in the long-form questionnaire. Therefore, the primary selection criterion for our analytical sample was the completion of this long-form survey, which inherently included older adults who reported any degree of disablement on the ADL or IADL scales. This process resulted in an analytical sample of 3,733 older adults. Subsequently, missing data on specific control variables (primarily life satisfaction and household type) within this analytical sample were handled using listwise deletion, yielding a final sample of 3,488 for the primary regression analyses.

### Variables

**Explained variable**: The explained variable is a composite index of disability risk, constructed from two validated instruments within the CLASS questionnaire: the Activities of Daily Living (ADL) scale and a cognitive function assessment. The ADL scale assesses difficulty in performing six basic activities (bathing, dressing, toileting, transferring, continence, feeding) and seven instrumental activities (IADL) (e.g., doing housework, managing money, taking medication). Responses were recorded on a 3-point scale: 1 = “No difficulty”, 2 = “Some difficulty”, 3 = “Cannot do”. A total ADL/IADL score was calculated, with higher scores indicating greater functional limitation. Cognitive function was assessed using a battery of questions testing general social knowledge, time orientation, and mathematical logic. The total score was standardized. Existing studies generally use the ADL/IADL scale to measure the risk of disability in older adults [20], given that cognitive decline is an important predictor of disability in older adults [21, 22], cognitive function can serve as a metric for assessing the risk of disability in older adults. To create a unified “disability risk” index, we first reversed the cognitive score so that higher values indicated poorer cognitive function, aligning its direction with the ADL score. We then standardized both the ADL and the reversed cognitive scores. The CRITIC (Criteria Importance Through Intercriteria Correlation) method was applied to assign objective weights to these two standardized components, resulting in a final weight of 0.339 for ADL and 0.661 for cognitive function. The composite disability risk score is the weighted sum of these two standardized components. Higher values on this continuous index indicate a higher risk of disability.

**Explanatory variables**: The explanatory variables are the respondents’ use of smart health devices. The survey asked, “Do you currently use any of the following smart health devices?” and provided a list. The devices included are all consumer-grade (non-prescription) devices: smart wheelchairs, electronic blood pressure monitors, blood lipid testers, smart bracelets/watches, smart cameras, smart all-in-one machines (e.g., devices integrating health monitoring with entertainment), smart sleep monitors, and audiobook players (see Table 2 for full list). “Use of smart health devices” is a binary variable (1 = uses at least one device; 0 = uses none). “Number of smart health devices” is a count variable (0–8) summing the total types of devices owned by the respondent.

#### Control variables

The study incorporates control variables, including gender (1 for male, 0 for female), age, ethnicity (1 for Han Chinese, 0 for ethnic minorities), marital status (1 for married, 0 for others), religious beliefs (1 for having beliefs, 0 for none), political affiliation (1 for party members, 0 for others), household size, household type (0 for rural, 1 for urban), and life satisfaction (measured on a 5-point Likert scale).

#### Mediating variables

The mediating variables encompass exercise frequency, participation in community rehabilitation therapy services, intergenerational financial support from children, and intergenerational domestic support. Exercise frequency was measured on a 9-point scale in response to the question, “How often do you engage in physical exercise or fitness activities?” with higher values indicating more frequent exercise (1 = least frequent, 9 = most frequent); Rehabilitation therapy is a binary variable (1 for participation, 0 for non-participation) based on the question, “In the past year, have you participated in any community-based rehabilitation therapy services?”; Intergenerational financial support is a binary variable (1 for receiving support, 0 for no support) from the question, “In the past year, have you received financial support from your children?”; Intergenerational domestic support was measured on a 5-point scale assessing the level of assistance with household chores received from children, with higher values indicating a greater degree of support (1 = least support, 5 = most support).

#### Moderating variables

Moderating variables encompass comparative health (respondents’ self-perception of health in relation to their peers; higher values indicate better health), recent hospitalization (1 for hospitalization in the last two years, 0 for no hospitalization), and the number of chronic diseases the respondent has.

Table 1 presents the descriptive statistics for the primary variables. The results indicated that the majority of the interviewed older adults exhibited a reduced risk of disability, a finding that may be attributable to the fact that the majority of the interviewed older adults were relatively younger or that those with lower levels of disability were more likely to be interviewed. (It should be noted that the risk of disability inherently includes the level of disability, thereby indicating a positive correlation between the level of disability and the risk of disability.) A survey revealed that 46.6% of older adults possessed at least one smart health device, with an average of 0.84 devices per individual. This finding suggests that there is considerable potential for the widespread adoption of smart health devices. A survey of older adults revealed that 46.6% of respondents reported ownership of at least one smart health device, with an average of 0.84 devices per person.

**Table 1:** Descriptive statistics of the main variables

Variables	Sample size	Std	Mean	Median	Min	Max
**Explained variable**						
Risk of disability	3733	0.186	-0.444	-0.496	-0.661	0.339
**Explanatory variables**						
Use of smart health devices	3733	0.499	0.466	0	0	1
Number of smart health devices	3733	1.207	0.840	0	0	8
**Control variables**						
Gender	3733	0.498	0.452	0	0	1
Age	3733	7.300	74.630	74	60	98
nation	3733	0.232	0.943	1	0	1
Marriage	3733	0.474	0.659	1	0	1
Religious belief	3733	0.254	0.069	0	0	1
Political status	3733	0.204	0.044	0	0	1
Household size	3733	1.360	2.922	2	1	10
Population	3540	0.496	0.433	0	0	1
Life satisfaction	3678	1.019	3.556	4	1	5

Table 2 presents the prevalence of smart health devices. The prevalence rate for electronic blood pressure monitors was found to be 43.1%, while the prevalence rate for lipid detectors was 11.4%. A study conducted in Chengdu revealed that the prevalence of hypertension among adults aged 80 and above reached an alarming 76.9% [23], while studies n the Changning District of Shanghai revealed that 59.9% of older adults aged 65 and above were affected by hypertension. However, only 32.8% of these individuals received treatment, and the blood pressure control rate was as low as 43.4% [24]; nationwide, the prevalence of hyperlipidemia among the older adults was 60.1%. Nationwide, the prevalence of hyperlipidemia among the older adults is 60.1%, with the highest prevalence of 85.6% among those aged 60 and older [25].

**Table 2:** Penetration rate of smart health devices

Variables	Sample size	Std	Mean	Median	Min	Max
Intelligent wheelchair	3733	0.265	0.076	0	0	1
sphygmomanometer	3733	0.495	0.431	0	0	1
Blood Lipid Tester	3733	0.318	0.114	0	0	1
Smart Bracelet/Watch	3733	0.289	0.092	0	0	1
Smart camera	3733	0.154	0.024	0	0	1
Intelligent All-in-One Machine	3733	0.203	0.043	0	0	1
Smart Sleep Monitor	3733	0.154	0.024	0	0	1
Recording of a person reading the text of a book	3733	0.186	0.036	0	0	1

### Models

The core analysis uses a multiple linear regression model. All control variables (gender, age, ethnicity, etc.) were entered simultaneously into the model. Categorical control variables (gender, ethnicity, marital status, religious belief, political status, household type) were effects-coded (0/1). The mediation analysis was conducted using the Sobel test and bootstrapping with 500 resamples to estimate indirect effects and 95% confidence intervals. For the moderation analysis, interaction terms were created by multiplying the mean-centered explanatory variable (device use/number) with each mean-centered moderating variable (comparative health, recent hospitalization, number of chronic diseases). These product terms were then added to the base regression model. The core explanatory variable of this study is the level of disability of the older adults, which is a continuous variable. To this end, the present study employed a multiple linear regression model to examine the relationship of smart health device use on the mental health level of older adults.

1 $$\:\:{Disability\_risk}_{i}=\alpha\:+{\beta\:equipment\_use}_{i}+{\delta\:Z}_{i}+{\varepsilon\:}_{i}$$

2 $$\:\:{Disability\_risk}_{i}=\alpha\:+{\beta\:equipment\_number}_{i}+{\delta\:Z}_{i}+{\varepsilon\:}_{i}$$

In Eq. (1) and Eq. (2), $\:{Disability\_risk}_{i}$ denotes the risk of disability of the older adults, $\:\alpha\:$ is a constant term, $\:{equipment\_use}_{i}$ denotes the use of smart health devices by the older adults (whether they have them or not), $\:{equipment\_number}_{i}$ denotes the number of smart health devices owned by the older adults (how many they have), $\:{Z}_{i}$ denotes a series of control variables, $\:\alpha\:$, $\:\beta\:$, and $\:\delta\:$ are the parameters to be estimated, and $\:{\epsilon\:}_{i}$ is a randomized perturbation term.

3 $$\:{X}_{ij}=\frac{{X}_{ij}-{X}_{min}}{{X}_{max}-{X}_{min}}$$

Since several variables in this study were assigned using the CRITIC assignment method, the CRITIC assignment method is described next. Firstly, the raw data are normalized according to Eq. (3), and if it is a negative indicator, the direction is converted using 1-$\:{X}_{ij}$. Where$\:{X}_{max}$ and $\:{X}_{min}$ is the maximum and minimum value of the *j*th indicator respectively, and $\:{X}_{ij}$ represents the data after standardization treatment. After the standardization process, the larger the value of all indicators, the better the situation.

4 $$\:{C}_{j}=\sigma\:{\mathrm{∑}}_{i=1}^{m}(1-|{r}_{ij}\left|\right)$$

5 $$\:{W}_{j}=\frac{{c}_{j}}{{\sum\:}_{j=1}^{n}{c}_{j}}$$

First, the information of the *j*th indicator $\:{C}_{j}$ is calculated according to Eq. (4), then the weight of the *j*th indicator $\:{W}_{j}$ is calculated according to Eq. (5), and finally all indicators are assigned according to the weight.

6 $$\:ATT=E\left\{E\right[{Y}_{1i}|{D}_{i}=1,P({X}_{i}\left)\right]-E\left[{Y}_{0i}\right|{D}_{i}=0,P\left({X}_{i}\right)\left]\right\}$$

This study uses propensity score matching method for robustness testing. The treatment group (above the mean) and control group (below the mean) are set according to whether the independent variable is above the mean. In Eq. (6), $\:{Y}_{1i}$ denotes the results of the treatment group and $\:{Y}_{0i}$ denotes the results of the control group. $\:{D}_{i}=1$ denotes the treatment group and $\:{D}_{i}=0$ denotes the control group. $\:P\left({X}_{i}\right)$ denotes the propensity score, and the logit model was used for propensity score matching.

The present study’s analytical plan was guided by a pre-specified theoretical framework, with hypotheses H1 through H5 representing the primary, confirmatory tests. The analyses pertaining to these hypotheses (i.e., the overall effect of device use/number, mediation, and moderation) are considered the core focus of this investigation. Subsequent analyses examining the effects of specific device types (Table 4) are exploratory in nature, intended to provide preliminary insights for future research rather than to test our central theoretical propositions.

### Empirical analysis

#### Baseline regression

As illustrated in Table 3, the results of the benchmark regressions are reported, which analyze the association of smart health devices on the risk of disability in older adults. Among them, model (1) and model (3) are the results of the benchmark regression without adding control variables. The association of smart health device use and the number of smart health devices with older adults’ risk of disability are represented by model (3), respectively. The findings indicate that, in comparison with older adults who do not utilize smart health devices, the risk of incapacitation among older adults who employ smart health devices is, on average, lower by 0.028. Furthermore, the risk of incapacitation is, on average, lower by 0.009 for each additional smart health device used, thereby providing evidence consistent with Hypothesis 1. The second and fourth models are the effects of smart health device use and the number of smart health devices on the risk of disability among older adults. After adding control variables, the results are basically consistent with the baseline regression. The magnitude of the association of smart health device utilization on the risk of disability among older adults is mitigated after the incorporation of control variables, while the influence of the number of smart health devices on the risk of disability among older adults not only does not diminish, but exhibits a slight increase following the addition of control variables. The regression results of models (5) and (6) were obtained through a robustness test that involved a reduction of the sample. Given that the risk of incapacitation among younger-aged older adults is considerably lower than that observed among middle-aged and high-aged older adults, the study’s sample was narrowed to include only older adults aged 70 and above for the purpose of conducting a robustness test. The findings indicate that the regression outcomes following the reduction of the sample are consistent with the conclusions of the benchmark regression, thereby substantiating the robustness of the conclusions of this study. Concurrently, the regression coefficients demonstrated minimal variation, indicating the absence of significant age heterogeneity in the negative association of smart health devices on the risk of disability in older adults. To contextualize the magnitude of these associations, we note that the composite disability risk index spans a range of 1.0 (from a minimum of -0.661 to a maximum of 0.339). Therefore, the coefficient of -0.021 for device use indicates an average reduction in disability risk equivalent to 2.1% of the scale’s total range, and each additional device is associated with a further 1.0% reduction. This provides a meaningful metric for interpreting the practical significance of the findings.

**Table 3:** Benchmark regression

Variables	(1)	(2)	(3)	(4)	(5)	(6)
Equipment use	-0.028***	-0.021***			-0.021***	
	(0.006)	(0.006)			(0.007)	
Number of equipment			-0.009***	-0.010***		-0.010***
			(0.002)	(0.002)		(0.003)
Gender		-0.016***		-0.016***	-0.020***	-0.020***
		(0.006)		(0.006)	(0.007)	(0.007)
Age		0.007***		0.007***	0.008***	0.008***
		(0.000)		(0.000)	(0.001)	(0.001)
Ethnic group		0.046***		0.047***	0.052***	0.052***
		(0.011)		(0.011)	(0.013)	(0.013)
Marriage		-0.033***		-0.033***	-0.037***	-0.037***
		(0.007)		(0.007)	(0.008)	(0.008)
Religious belief		0.031***		0.029***	0.034***	0.032**
		(0.011)		(0.011)	(0.013)	(0.013)
Political status		-0.002		-0.001	-0.010	-0.009
		(0.011)		(0.011)	(0.016)	(0.016)
Household size		0.013***		0.014***	0.015***	0.015***
		(0.002)		(0.002)	(0.002)	(0.002)
Population		-0.038***		-0.038***	-0.040***	-0.038***
		(0.006)		(0.006)	(0.008)	(0.007)
Life satisfaction		-0.041***		-0.041***	-0.048***	-0.048***
		(0.003)		(0.003)	(0.003)	(0.003)
Constant term	-0.431***	-0.883***	-0.436***	-0.893***	-0.924***	-0.934***
	(0.004)	(0.036)	(0.004)	(0.036)	(0.055)	(0.055)
N	3733	3488	3733	3488	2505	
R^2^	0.0055	0.2078	0.0037	0.2085	0.1973	0.1982

Note: *, **, and *** indicate significant at the 10%, 5%, and 1% levels, respectively, with robust standard errors in parentheses, below

As illustrated in Table 4, the investigation explores the association between the utilization of diverse categories of smart health devices and the risk of disability in older adults. The models include the following: model (1) is a smart wheelchair, model (2) is an electronic blood pressure monitor, model (3) is a lipid detector, model (4) is a smart bracelet/watch, model (5) is a smart camera, model (6) is a smart all-in-one machine, model (7) is a smart sleep monitor, and model (8) is an audio book. The findings indicated that the utilization of smart wheelchairs was associated with a significant positive correlation with the risk of disability in older adults. This association can be primarily attributed to the fact that older adults who do not require a wheelchair do not need to use one. Furthermore, a comparison of older adults who use a smart wheelchair with those who do not use a smart wheelchair in isolation may reflect the attenuating effect of using a smart wheelchair on the risk of disability in older adults who already require a wheelchair. The utilization of electronic blood pressure monitors, lipid meters, smart bracelets/watches, and smart all-in-one computers has been demonstrated to result in a substantial reduction in the risk of disability among older adults. The smart bracelet/watch demonstrated the most significant effect value, followed by the smart all-in-one and the lipid detector. A distinguishing characteristic of smart bracelets and watches, when contrasted with other smart health devices, is their capacity to continuously monitor the health status of older adults and assist them in managing their health. Concurrently, smart bracelets/watches frequently possess the capacity to monitor multiple health indicators (e.g., blood glucose, heart rate, etc.). Moreover, they can function as a medium for communication, facilitating connections between older adults and their children or friends, thereby fostering social support and emotional exchange. This likely contributes to their optimal efficacy in reducing the risk of disability among older adults. In addition to facilitating communication for older adults, the primary function of smart all-in-one devices is to provide recreation and entertainment. The utilization of smart devices for leisure purposes has been demonstrated to enhance the cognitive abilities of older adults [26]. This enhancement has been shown to mitigate cognitive decline and, consequently, minimize the likelihood of disability in this demographic.

**Table 4:** Exploratory analysis: association between different types of smart health device use and disability risk in older adults

Variables	(1)	(2)	(3)	(4)	(5)	(6)	(7)	(8)
Equipment use	0.051***	-0.018***	-0.050***	-0.054***	0.005	-0.052***	-0.002	-0.007
	(0.012)	(0.006)	(0.008)	(0.008)	(0.017)	(0.010)	(0.016)	(0.012)
Gender	-0.015***	-0.016***	-0.016***	-0.016***	-0.015***	-0.015***	-0.015***	-0.015***
	(0.006)	(0.006)	(0.006)	(0.006)	(0.006)	(0.006)	(0.006)	(0.006)
Age	0.007***	0.007***	0.008***	0.007***	0.007***	0.007***	0.007***	0.007***
	(0.000)	(0.000)	(0.000)	(0.000)	(0.000)	(0.000)	(0.000)	(0.000)
Ethnic group	0.046***	0.048***	0.044***	0.042***	0.048***	0.047***	0.048***	0.048***
	(0.011)	(0.011)	(0.011)	(0.011)	(0.011)	(0.011)	(0.011)	(0.011)
Marriage	-0.033***	-0.033***	-0.033***	-0.034***	-0.034***	-0.033***	-0.034***	-0.034***
	(0.007)	(0.007)	(0.007)	(0.007)	(0.007)	(0.007)	(0.007)	(0.007)
Religious belief	0.032***	0.031***	0.029***	0.027**	0.031***	0.031***	0.031***	0.031***
	(0.011)	(0.011)	(0.011)	(0.011)	(0.011)	(0.011)	(0.011)	(0.011)
Political status	-0.007	-0.003	-0.005	0.000	-0.006	-0.004	-0.006	-0.005
	(0.011)	(0.011)	(0.011)	(0.011)	(0.011)	(0.011)	(0.011)	(0.011)
Household size	0.011***	0.013***	0.013***	0.014***	0.012***	0.013***	0.012***	0.012***
	(0.002)	(0.002)	(0.002)	(0.002)	(0.002)	(0.002)	(0.002)	(0.002)
Population	-0.051***	-0.039***	-0.040***	-0.040***	-0.046***	-0.042***	-0.046***	-0.046***
	(0.006)	(0.006)	(0.006)	(0.006)	(0.006)	(0.006)	(0.006)	(0.006)
Life satisfaction	-0.041***	-0.042***	-0.040***	-0.040***	-0.041***	-0.041***	-0.041***	-0.041***
	(0.003)	(0.003)	(0.003)	(0.003)	(0.003)	(0.003)	(0.003)	(0.003)
Constant term	-0.864***	-0.885***	-0.899***	-0.892***	-0.886***	-0.882***	-0.887***	-0.886***
	(0.037)	(0.036)	(0.036)	(0.036)	(0.036)	(0.036)	(0.036)	(0.036)
control variables	be							
N	3488							
R^2^	0.2103	0.2071	0.2126	0.2121	0.2051	0.2086	0.2051	0.2051

### Propensity score matching

**Fig. 2:**
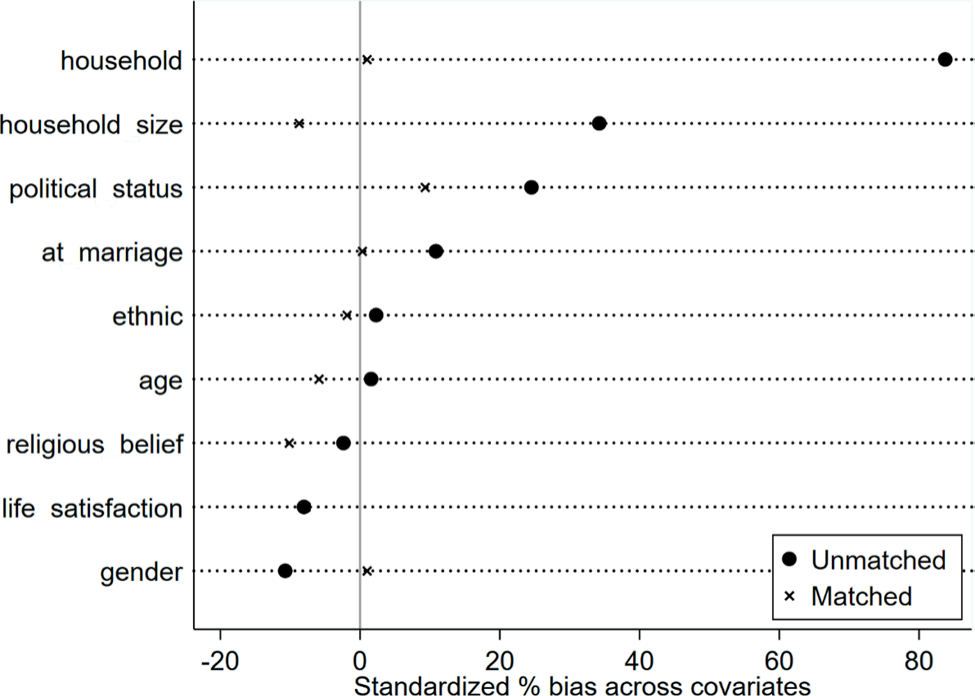
Change in standard deviation of covariates after nearest neighbor matching

Propensity Score Matching (PSM) can be used in social research to estimate the causal effects of an intervention or policy by balancing the distributions of observable characteristics of the treatment and control groups to simulate randomized experimental conditions [27] .As illustrated in Table 5, the mean treatment effects of smart health device use are reported to demonstrate the potential for mitigating the risk of disability. Figure 2 provides a visual representation of the variation in standard deviation of the near-neighbor matching candidate covariates. The findings indicate that the mitigating association of smart health device utilization on the risk of incapacitation among older adults surpasses the propensity score value matching of most methods, and the standard deviation of most covariates after matching does not deviate from the 0 value by more than 10% points, suggesting that the primary conclusions of this study withstand the robustness test.

**Table 5:** Mean treatment effects of smart health device use weakening the risk of disability

Matching method	brochure	ATT	standard error	t-value
nearest neighbor matching (1:3)	unmatch	-0.026***	0.006	-4.23
	match	-0.019**	0.009	-2.14
nuclear matching	unmatch	-0.026***	0.006	-4.23
	match	-0.012	0.008	-1.57
radius match	unmatch	-0.026***	0.006	-4.23
	match	-0.026***	0.004	-5.78

### Heterogeneity analysis

The term “digitally marginalized” in the title and text refers to older adults characterized by two key dimensions: lack of digital access and low digital literacy. Digital access was measured by the question, “Do you have access to the internet at home or via a mobile device?” (0 = No; 1 = Yes). Digital literacy was operationalized as proficiency in using internet-enabled devices. Respondents who reported being proficient in using such devices were classified as “practiced” (1), while those who reported not being proficient were classified as “unskilled” (0). For heterogeneity analysis, the sample was stratified by these variables to define subgroups facing the digital divide (no access) and having low digital literacy (unskilled).

Table 6 presents the heterogeneous impacts of smart health devices on the disability risks of older adults. The findings suggest that the advantages are more pronounced for individuals experiencing the digital divide, potentially due to their disproportionately poorer health status. This outcome stands in contrast to the predictions derived from the technology acceptance model, which anticipated that digitally literate users would derive greater benefits. This underscores the intricate relationship between health inequality and smart aging.

**Table 6:** Heterogeneity analysis

Variables	Digital divide heterogeneity				Digital literacy heterogeneity			
	digital divide		digital access		unskilled		practiced	
Equipment use	-0.017**		-0.018		-0.029		-0.019***	
	(0.007)		(0.013)		(0.019)		(0.006)	
Number of equipment		-0.008***		-0.005*		-0.009*		-0.009***
		(0.003)		(0.003)		(0.005)		(0.003)
constant term	-0.845***	-0.854***	-0.751***	-0.773***	-0.810***	-0.816***	-0.877***	-0.888***
	(0.043)	(0.043)	(0.078)	(0.079)	(0.159)	(0.161)	(0.037)	(0.038)
control variables	be	be	be	be	be	be	be	be
N	2862		626		267		3221	
R^2^	0.1830	0.1833	0.1203	0.1200	0.1019	0.1012	0.2116	0.2123

### Analysis of mechanisms

#### Intermediary mechanisms

According to the findings presented in the theoretical hypotheses section, this study employed the Sobel method to calculate the mediating effect share of the mediating variables. Additionally, the bootstrap method was utilized to determine the confidence intervals and assess the robustness of the findings. The results of the mediating effect analysis are reported in Table 7. The findings indicate that the negative association between smart health devices and disability risk can be partially explained by their positive correlation with increased physical activity, enhancing access to community-based rehabilitation services, and facilitating the acquisition of intergenerational financial and household assistance. These outcomes substantiate Hypothesis 2a, Hypothesis 2b, Hypothesis 3a, and Hypothesis 3b, thereby underscoring the significance of these factors in promoting health and well-being in older populations. The effect that accounted for the largest proportion of the results was frequency of exercise, which accounted for 34.03%. Moderate- to high-intensity exercise, such as a 12-week systematic program, has been shown to significantly enhance muscle strength, functional capacity, and balance. These benefits contribute to the retardation of physical function decline and the mitigation of the risk of disability due to muscle weakness or falls [28]. Regular exercise has been demonstrated to reduce the risk of several chronic diseases, including type 2 diabetes (40% risk reduction), cardiovascular disease (35% reduction), joint pain (25% reduction), and falls (more than 30% reduction). These conditions are significant contributors to disability in older adults [29]. Consequently, the engagement in physical activity can serve as a mitigating factor in the development of disability among older adults. According to behavioral change theory, smart health devices can assist older adults in comprehending their health status with greater precision. Moreover, smart health devices, including smart bracelets and watches, have the potential to motivate older adults to engage in physical activity and document their exercise data. Consequently, smart health devices can encourage older adults to participate in exercise more frequently, thereby reducing the likelihood of incapacitation.

Social support (including intergenerational support) significantly improves the mental health status of older adults, and informal social support (e.g., emotional care from children) reduces depressive symptoms [30], and relief of depressive symptoms is associated with improved cognitive function [31]. The presence of depressive symptoms has been demonstrated to increase the risk of disability. Intergenerational social support has been shown to reduce the risk of disability in older adults. The provision of financial support by children has been demonstrated to exert a substantial positive influence on the physical and mental well-being of older adults, particularly with regard to mental health [32]. For instance, financial assistance can facilitate the acquisition of superior medical resources, nutritional provisions, or assistive devices, all of which are directly associated with the preservation of physical functionality. The correlation between the physical and mental health conditions of older adults and the risk of incapacitation is well-documented. Smart health devices have the potential to reduce the risk of incapacitation by helping older adults access intergenerational social support.

**Table 7:** Analysis of intermediation effects

Form	Intermediary variables	Bootstrap 95% confidence interval	Indirect benefits	Efficiency ratio
Utilization equipment	Exercise frequency	[-0.0092601, -0.0047336]	-0.0070***	34.03%
			(0.0012)	
	rehabilitation	[-0.0024381, -0.0005485]	-0.0015***	7.26%
			(0.0006)	
	Intergenerational economic support	[-0.002845, -0.000141]	-0.0015**	7.26%
			(0.0006)	
	Intergenerational domestic support	[-0.0042026, -0.0005686]	-0.0024***	13.69%
			(0.0009)	
Number of equipment	Exercise frequency	[-0.0029575, -0.0011255]	-0.0020***	21.34%
			(0.0004)	
	rehabilitation	[-0.0014643, -0.0003449]	-0.0009***	9.45%
			(0.0003)	
	Intergenerational economic support	[-0.0009712, -0.0001021]	-0.0005**	5.61%
			(0.0002)	
	Intergenerational domestic support	[-0.0015625, -0.0002403]	-0.0009***	10.98%
			(0.0003)	

### Regulatory mechanisms

**Fig. 3:**
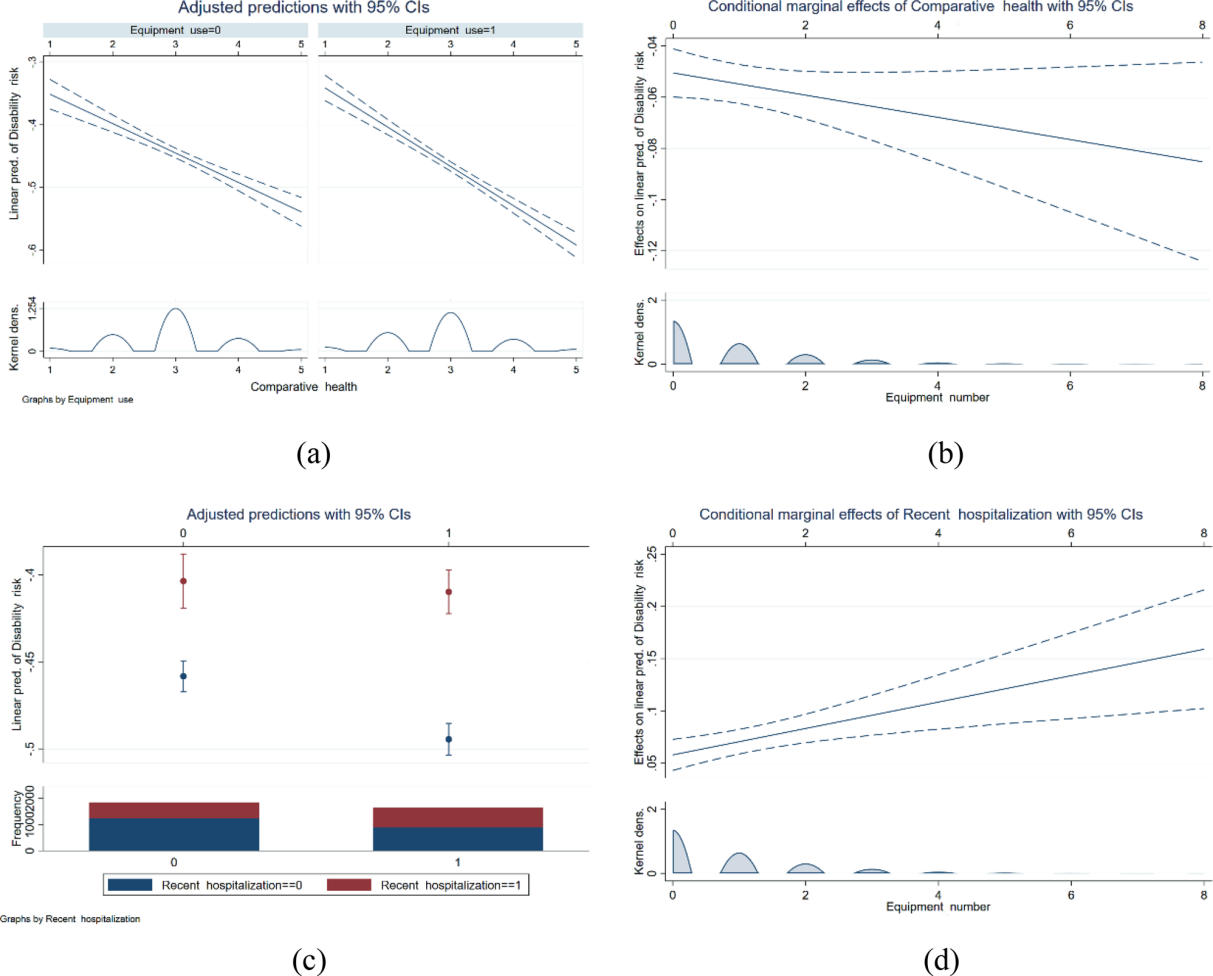
Diagram of modulation effect

In accordance with the theoretical framework, this study incorporated cross-terms into regression models to test moderating effects. The results of the moderating effects analysis are presented in Table 8. The findings indicate that comparative health significantly weakens the negative association of smart health devices on older adults’ disability risk (statistically significant at the 1% level), thereby supporting Hypothesis 4a. The correlation between comparative health and socioeconomic status is such that individuals from lower economic backgrounds tend to have less access to healthcare resources and consequently benefit less from the use of smart health devices. According to the principles of health capital theory, an individual’s current health status is a reflection of their past health investments. Consequently, a decrease in health capital would indicate a reduction in historical health investments. Given that the utilization of smart devices can be regarded as a health investment, comparative health moderates the relationship between device disability. As demonstrated in Fig. 3(a-b), this moderation is evident in the amplified negative association of comparative health on smart health device usage and quantity.

The findings presented in Table 8 demonstrate a substantial reinforcing effect of recent hospitalization on the attenuating effect of smart health devices on the risk of disability among older adults. This effect is deemed to be statistically significant at the 1% confidence level, thereby validating Hypothesis 4b. A considerable number of older adults exhibit reluctance to seek hospitalization, despite the presence of health concerns, with the primary motivation being financial conservatism [33]. According to the principles of health capital theory, the propensity of older adults to seek hospitalization is indicative of a heightened readiness to allocate financial resources toward their health and well-being. This positive mindset has been shown to facilitate the acceptance of new technologies and smart health devices, which in turn has been demonstrated to reduce the risk of disability. The moderating effect of recent hospitalization is more significant in cases involving a higher number of smart health devices. However, the number of chronic diseases in older adults did not significantly moderate the association of smart health devices on reducing incapacitation risk, thus falsifying hypothesis 4d.

**Table 8:** Analysis of moderating effects

Variables	Risk of disability					
Use of smart health devices	-0.027***		-0.027***		-0.020***	
	(0.006)		(0.006)		(0.006)	
Number of smart health devices		-0.012***		-0.014***		-0.011***
		(0.003)		(0.002)		(0.002)
Compare Health× Device Use	-0.027***					
	(0.007)					
Compare Health× Number of devices		-0.009***				
		(0.003)				
Recent Hospitalizations× Equipment Use			0.047***			
			(0.012)			
Recent hospitalizations× Number of devices				0.018***		
				(0.004)		
Number of chronic diseases× Equipment use					-0.001	
					(0.004)	
Number of chronic diseases× Number of devices						0.002
						(0.001)
Control variables	be	be	be	be	be	be
R^2^	0.2135	0.2141	0.2117	0.2122	0.2078	0.2091
N	3467		3488		3488	

## Discussion

Our findings illuminate the complex relationship between smart health devices and disability risk among older adults in China and directly address the conceptual and methodological gaps identified in the extant literature. Recall that prior studies have often focused on single devices in isolation, lacked large-scale empirical tests of the direct association, and left the underlying mechanisms—the how and for whom—underexplored. By employing a national dataset and testing an integrated theoretical framework, this study provides a more unified analysis. Moving beyond a simple cause-effect narrative, the following discussion interprets the results through the lens of HBM, Social Support Theory, HCT, and TAM to explicitly explain the pathways and contingencies our model revealed. The observed associations, while statistically significant, are modest in absolute terms. As indicated by our results, smart health device use is associated with a reduction in disability risk of 2.1% on the composite scale. To assess the substantive importance of this effect, we benchmark it against other correlates in our model. This 2.1% association is comparable to the protective association of being married (-3.3%) and is larger than the difference associated with gender (-1.6%). This suggests that the association of smart device use is not trivial when contextualized against other well-established demographic factors. However, the primary public health significance may lie not in the individual-level effect but in its potential for population-level impact. Given the vast and growing population of older adults in China, a small shift in the average population risk, if causal and scalable, could translate into a substantial number of disability cases prevented.

A finding with significant implications for both theory and policy is that the disability risk reduction associated with smart health devices was more pronounced for older adults experiencing a digital divide and those with lower digital literacy. From a pure Technology Acceptance Model (TAM) perspective, this appears counter-intuitive, as higher perceived ease of use and usefulness—typically correlated with greater digital access and literacy—are expected to lead to better outcomes [19]. Our results, however, suggest a more complex dynamic that refines the application of TAM in contexts of social and health inequality. We posit that for digitally marginalized older adults, who may face compounded health disadvantages and have fewer alternative health management resources, the marginal gain from successfully adopting a useful technology is substantially higher. A smart health device might represent their first or only structured form of health monitoring and support, thus having a greater absolute impact on reducing disability risk. In contrast, for older adults with robust digital access and literacy, these devices may be one component in a broader portfolio of health resources, resulting in a smaller observable marginal effect. This finding is a key conceptual contribution, highlighting that the perceived usefulness in TAM is not static but is relative to an individual’s existing health capital and resource portfolio. It suggests that interventions targeting digital marginalization are not merely about equity in access but are crucial for maximizing population-level health gains, as these groups stand to benefit the most from successful technology adoption.

The mediating roles of exercise frequency, rehabilitation use, and intergenerational support strongly align with the pathways hypothesized by HBM and Social Support Theory. As HBM suggests, devices acted as powerful cues to action, making abstract health risks tangible and motivating engagement in proactive behaviors like exercise (H2a) [16]. The finding that exercise was the strongest mediator underscores the centrality of physical activity in mitigating functional decline. Similarly, the results for intergenerational financial and household support (H3a, H3b) corroborate the buffering model of Social Support Theory [17]. In the Chinese familial context, smart devices serve as a technological bridge, facilitating the flow of support from adult children to their aging parents. By providing remote, concrete data on a parent’s health status, these devices likely trigger more targeted and timely financial and instrumental interventions, thereby reducing disability risk. The non-significant effect of smart cameras further enriches this discussion, suggesting that technology which potentially replaces, rather than facilitates, meaningful human interaction may be less effective, emphasizing the irreplaceable value of offline, empathetic care in Confucian cultures [34, 35].

Our moderating findings offer nuanced support for HCT. The positive moderating effect of recent hospitalization (H4b) aligns with HCT’s investment logic: individuals who have recently invested in their health via hospitalization may be more receptive to further investments like smart devices, amplifying the devices’ benefits [17]. However, the negative moderating effect of comparative health (H4a) and the non-significant effect of chronic diseases (H4c) present a more complex picture. It appears that while a willingness to invest (captured by hospitalization) strengthens the device-disability relationship, simply having low health capital (inferred from poor comparative health) is not a consistent moderator. This suggests that the decision to use a device effectively is not merely a function of health stock but is also mediated by psychological factors and socioeconomic resources that enable investment, which were not fully captured by our measures. Future research should explore the interplay between objective health capital and the subjective propensity to invest in it.

### Theoretical implications, contextual strengths, and limitations

This study demonstrates both the value and the challenge of applying a multi-theoretical framework in a specific socio-cultural context. The integration of TAM, HBM, Social Support Theory, and HCT provided a more holistic “story” than any single theory could, explaining not only the “how” but also the “for whom” and “under what conditions”. A key theoretical implication is the need to contextualize technology adoption models like TAM within frameworks of social inequality. The finding that the most marginalized benefit most suggests that theories must account for marginal utility and pre-existing resource disparities, not just adoption drivers. A particular strength of our study in the Chinese context was the explicit modeling of intergenerational support, a cornerstone of the local culture, as a critical mechanism. This moves beyond Western-centric individual-level models and demonstrates how technology can integrate with, and strengthen, existing familial support structures. Furthermore, our focus on the digital divide directly addresses a pressing issue in China’s rapid technological modernization.

However, several limitations must be acknowledged. First, the cross-sectional nature of the CLASS2020 data prevents definitive causal claims about the mediating pathways. While our theoretical model and analytical methods are robust, longitudinal or experimental designs are needed to firmly establish the causal chain from device use, through mediators, to disability risk. Second, our measures, while derived from a high-quality national dataset, are necessarily simplified. For instance, “digital literacy” is a complex, multi-dimensional construct that our binary measure could not fully capture. Future studies would benefit from more nuanced scales that assess not just competence but also digital confidence and learning preferences. Third, given our paper’s emphasis on digital marginalization and psychosocial mechanisms like social support, we did not fully explore the role of peer-based digital environments. Fourth, the observed associations, while statistically significant, represent a reduction of 2.1% on the disability risk scale. The clinical meaningfulness or minimal clinically important difference for this specific composite measure is not yet established, and the individual-level benefit should be interpreted with this context in mind. Finally, it is important to acknowledge that the study involved multiple statistical comparisons, particularly in the exploratory analysis of individual device types. While our primary conclusions are based on a pre-specified theoretical model and supported by robustness checks, the potential for inflated Type I error across the full set of tests should be considered. Future research with pre-registered hypotheses would be valuable to confirm these findings. Beyond the intergenerational support modeled here, future research could investigate how older adults and their caregivers engage with online communities and peer platforms to enhance motivation, confidence, and sustained adherence in using smart health technologies. These platforms may serve as vital informal learning spaces that complement formal device usage and reinforce health-related behaviors through social learning and shared experiences, potentially mitigating the negative effects of digital marginalization [36]. Finally, the generalizability of our integrated model to other cultural contexts with different family structures and welfare systems remains to be tested.

## Conclusions

This study examined the association of smart health devices on the risk of disability in older adults in China. The investigation utilized the CLASS2020 dataset and employed multiple linear regression modeling techniques. The heterogeneity analysis indicated that the negative association between smart health devices and disability risk was more significant for older adults in the digital divide and those with good digital literacy. The statistical mediation analysis suggested that the relationship between smart health devices and disability risk can be partially explained by their correlation with higher levels of intergenerational support, greater physical activity, and increased rehabilitation utilization. Secondly, an analysis of the moderating effects indicated that the comparative health level negatively moderated the relationship, while recent hospitalization positively moderated the relationship. Thirdly, the present study investigated the combined association of multiple types of smart health devices on disability risk and distinguished the heterogeneity of different device types.

## Data Availability

Any reader interested in the study can contact zhangchi@lzu.edu.cn for data availability.
